# Thrombin inhibition and cisplatin block tumor progression in ovarian cancer by alleviating the immunosuppressive microenvironment

**DOI:** 10.18632/oncotarget.13300

**Published:** 2016-11-11

**Authors:** Eric T. Alexander, Allyson R. Minton, Molly C. Peters, Joanne van Ryn, Susan K. Gilmour

**Affiliations:** ^1^ Lankenau Institute for Medical Research, Wynnewood, PA 19096, USA; ^2^ Boehringer Ingelheim Pharma GmbH & Co. KG, 88397 Biberach an der Riss, Germany

**Keywords:** thrombin, immunosuppression, MDSCs, ovarian cancer, dabigatran

## Abstract

Cancer is often associated with an increased risk of thrombotic complications which can be aggravated by treatment with chemotherapeutics such as cisplatin. Multiple lines of evidence suggest that thrombin activity promotes tumor growth and metastasis. We examined the effect of co-treatment with dabigatran etexilate, a direct thrombin inhibitor, and cisplatin using the murine ID8 ovarian cancer model. Mice receiving co-treatment with both dabigatran etexilate and low dose cisplatin had significantly smaller tumors, developed less ascites and had lower levels of circulating activated platelets and tissue factor (TF) positive microparticles than those treated with dabigatran etexilate or cisplatin alone. Co-treatment with dabigatran etexilate and cisplatin significantly decreased the number of Gr1^+^/CD11b^+^ myeloid derived suppresser cells and CD11b^+^/CD11c^+^ dendritic cells in the ascites of ID8 tumor-bearing mice. Co-treatment also significantly reduced levels of pro-tumorigenic cytokines including TGF-β, VEGF, IL-6, IL-10, and MCP-1 in the ascites while increasing IFN-γ production by CD8^+^ effector T cells in the tumor ascites. These results demonstrate that co-treatment with dabigatran etexilate significantly augments the anti-tumor activity of cisplatin in ovarian tumor progression by alleviating the immunosuppressive microenvironment, suggesting that thrombin may be a potential therapeutic target for treatment of ovarian cancer.

## INTRODUCTION

The association between thrombosis and cancer dates back to 1865 when Armand Trousseau observed that patients who presented with idiopathic venous thromboembolism frequently had an underlying cancer. Epidemiological studies have shown that cancer patients are at elevated risk for developing venous thromboembolism (VTE) and pulmonary embolism [1] with up to 50% of all cancer patients exhibiting hemostatic abnormalities [2]. Unfortunately, treatment with standard cancer chemotherapeutic agents such as cisplatin exacerbates this risk [3]. Although the mechanisms are not clearly understood, chemotherapeutic agents have pro-thrombotic side effects associated with platelet activation, increased cellular exposure of phosphatidylserine (PS) and elevation of tissue factor (TF)-positive microparticles [4, 5].

Thrombin is the primary effector protease of the coagulation cascade generated by the action TF and other coagulation factors. The critical role of thrombin in promoting tumor growth reflects its many functions, including fibrin formation [6], platelet activation [7], activation of PAR signaling [8] and the proteolytic breakdown of extracellular matrix. Specifically, thrombin directly stimulates tumor cell adhesion, growth, DNA synthesis and cellular proliferation [9]. Thrombin generation also promotes metastasis through fibrin deposition, platelet activation and via PAR-1 signaling [10]. Thrombin is an effective promoter of angiogenesis by both clotting-dependent mechanisms, via platelet activation and fibrin deposition, and clotting-independent mechanisms mediated by PAR activation. Activated platelets further augment angiogenesis by releasing VEGF and platelet-derived growth factors [11, 12]. Finally, fibrin formation and platelet-derived TGF-β can inhibit natural killer cell activity, helping the tumor evade host immunosurveillance [13, 14].

Ovarian cancer has the highest mortality rate of all gynecological cancer worldwide [15]. Even with debulking surgery followed by front-line chemotherapy, advanced-stage disease is often incurable due to the development of chemoresistant disease which results in a 5-year survival rate of only 27% [16]. More than one third of ovarian cancer patients present with malignant ascites at diagnosis [16]. Development of ascites is a fundamental part of chemo-resistant disease [17]. Ascites development is associated with poor prognosis and deterioration in patient quality of life. Malignant ascites is a reservoir of proinflammatory and immunosuppressive cytokines, chemokines, growth factors and immune cells which generates a pro-tumorigenic microenvironment that facilitates tumor cell growth, suppression of the immune system, and resistance to standard chemotherapy [18].

Given that chemotherapy exacerbates the state of hyper-coagulation that promotes cancer progression, we hypothesized that inhibition of thrombin with dabigatran etexilate would act cooperatively with cisplatin, a front-line treatment of ovarian cancer, to inhibit tumor growth and the development of malignant ascites. Dabigatran etexilate is a new oral anticoagulant that is a direct thrombin inhibitor [19]. Using the ID8 murine model of ovarian cancer we showed significantly greater anti-tumor efficacy with dabigatran etexilate and cisplatin co-treatment that was accompanied by a decrease in immunosuppressive myeloid cell populations and pro-tumorigenic cytokines as well as a concomitant increase in CD8^+^ effector T-cell activity in the tumor ascites.

## RESULTS

### Dabigatran etexilate and cisplatin inhibit tumor growth and ascites development *in vivo*

**Figure 1:**
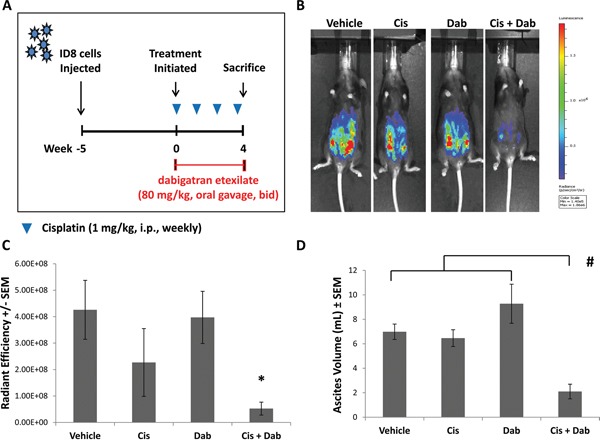
Inhibitory effects of cisplatin and dabigatran etexilate co-treatment on ID8 tumor growth and malignant ascites accumulation **A.** Schematic of ID8 ovarian tumor model. Five weeks after mice were i.p. injected with 1.0 x 10^6^ ID8-luc cells, treatment was initiated. Mice were injected i.p. with cisplatin (1-2 mg/kg) once weekly with or without dabigatran etexilate administration. Dabigatran etexilate was administered by oral gavage twice daily (80 mg/kg) Monday through Friday, and mice were placed on dabigatran chow (10 mg/g chow) over the weekends. **B.** Representative quantification of ID8-luc tumor burden by bioluminescence imaging in living mice. **C.** Upon sacrifice, the ascites fluid was collected, and the final tumor loads were assessed by bioluminescence imaging of the opened peritoneal cavity. **D.** Ascites volume was determined upon sacrifice. n = 5-10 mice per group. * = p<0.05 and # = p<0.01 compared to control vehicle-treated tumor bearing mice.

Because cancer often induces a pro-thrombotic state that is exacerbated by chemotherapeutic agents, we evaluated the effect of thrombin inhibition with dabigatran etexilate in conjunction with low dose chemotherapeutic treatment using the ID8-luc ovarian cancer model. Following i.p. injection of ID8-luc in female C57/Bl6 mice, treatment was initiated when tumor bioluminescence was approximately 5.0 x 10^5^ photons/sec/cm^2^. Mice were administered 1 mg/kg cisplatin i.p. once a week, with or without dabigatran etexilate administrated by oral gavage (80 mg/kg twice daily, Monday-Friday, dabigatran food (10 mg/g food pellets) on the weekends). Treatment with either low dose cisplatin treatment or dabigatran etexilate alone modestly reduced ID8 tumor growth compared to vehicle treatment as measured by bioluminescence (Figure 1B and 1C). However, there was a significant inhibitory effect on tumor growth in mice treated with both cisplatin and dabigatran etexilate with a 14-fold reduction in ID8 tumor spread as measured by final peritoneal bioluminescence compared to that in vehicle-treated mice. Co-treatment with cisplatin and dabigatran etexilate did not significantly reduce the proliferative index as determined by Ki67 immunostaining despite the decreased tumor load in mice co-treated with cisplatin and dabigatran etexilate (Supplementary Figure S1).

A common feature of ovarian cancer is the development of ascites, a fluid accumulation in the peritoneal cavity containing a dynamic mixture of tumor cells, growth factors and immune cells [18]. By 8 weeks following the injection of ID8-luc cells, pronounced ascites accumulation was observed (Figure 1D). Treatment with cisplatin or dabigatran etexilate individually did not affect the volume of ascites that developed. However, in mice treated with both cisplatin and dabigatran etexilate there was an approximately 3-fold reduction in ascites volume.

### Dabigatran etexilate treatment reduces platelet activation and inhibits the generation of tissue factor (TF) positive microparticles

**Figure 2:**
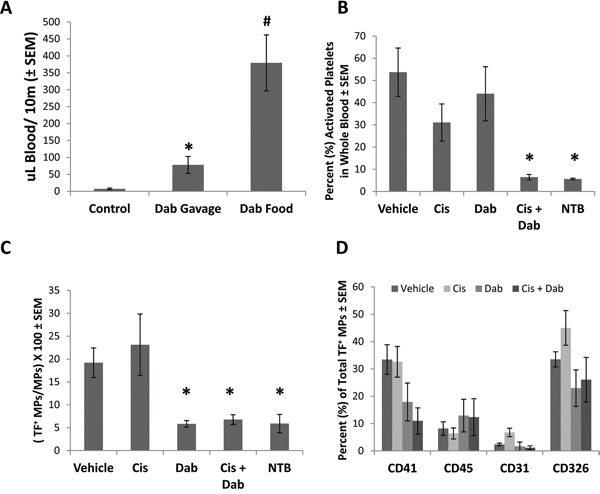
Cisplatin and dabigatran etexilate co-treatment reduces platelet activation and the generation of tissue factor-positive microparticles (TF^+^ MPs) Upon sacrifice, blood was collected from ID8 tumor bearing mice, or non-tumor bearing mice (NTB), and 50 μl was treated with ammonium oxalate to lyse red blood cells for platelet analysis. The remaining whole blood was sequentially spun to remove all cells, generating platelet poor plasma (PPP). **A.** Non-tumor bearing mice were given either a single dose of dabigatran etexilate (80 mg/kg) by oral gavage or placed on dabigatran chow (10 mg/g) for 48 h. After tail transaction, total blood loss over 10 minutes was quantified. **B.** Activated platelets were identified by flow cytometry. **C.** TF^+^ MPs in PPP were quantified by flow cytometry. **D.** Cell origin of TF^+^ MPs stained with anti-CD41 (platelets), anti-CD45 (leukocytes), anti-CD31 (endothelial cells) and anti-CD326 (epithelial cells) antibodies in PPP isolated from ID8 tumor bearing mice. n = 5-10 mice per group. * = p<0.05 and # = p<0.01 compared to control vehicle-treated tumor bearing mice.

Mice treated with dabigatran etexilate showed a substantial increase in total blood loss following tail transection (Figure 2A) indicating that inhibition of thrombin significantly impaired clotting. Activated platelets are a potent initiator of the clotting cascade which can be activated by thrombin. Platelets were analyzed in peripheral blood of ID8 tumor bearing mice following treatment with cisplatin ± dabigatran etexilate. The number of platelets per *u*l of blood was similar in all groups (data not shown). Platelets were assayed for activation by flow cytometric analysis after staining for CD41, a platelet marker, and CD62P (P-selectin) which is expressed upon platelet activation. Platelet activation (%) was markedly elevated in ID8-luc tumor bearing mice (Figure 2B) compared to non-tumor bearing mice. Treatment with cisplatin or dabigatran etexilate individually did not significantly reduce platelet activation, whereas co-treatment reduced the tumor induced increase in platelet activation to the levels found in non-tumor-bearing mice (Figure 2B).

Studies have demonstrated an increase in the number of circulating microparticles in cancer patients compared to healthy individuals [20], and that TF^+^ microparticles play an important role in systemic coagulopathies in cancer patients and possibly tumor progression and metastasis [21]. We previously demonstrated that dabigatran etexilate significantly reduced the number of circulating TF^+^ microparticles in mice with breast cancer [22]. To determine if dabigatran etexilate similarly reduced circulating TF^+^ microparticles in ovarian cancer, plasma from mice with ID8 ovarian tumors was analyzed. Vehicle treated mice with ID8 tumors showed a 4-fold increase in the percentage of circulating TF^+^ microparticles (Figure 2C). Treatment with cisplatin had no effect, but dabigatran etexilate, with or without cisplatin, completely prevented the tumor induced increase in circulating TF^+^ microparticles resulting in levels similar to non-tumor bearing mice (Figure 2C).

The composition of microparticles depends on their cell of origin, with microparticles generated by different cells containing different proteins, lipids and nucleic acids. Circulating TF^+^ microparticles in platelet poor plasma were stained for CD41 (platelets), CD45 (leukocytes), CD31 (endothelial cells) and CD326 (ID8 epithelial cells). However, the cellular origin of TF^+^ microparticles in non-tumor bearing mice could not be determined due to their very low number compared to ID8 tumor bearing mice.

Circulating TF^+^ microparticles in ID8 tumor bearing mice were primarily of platelet and tumor cell origin (Figure 2D) with 33.4 ± 5.4% and 33.5 ± 2.8% of the total TF^+^ microparticles staining for CD41 and CD326 respectively. Treatment with cisplatin alone did not alter the cellular origin of TF^+^ microparticles, but treatment with dabigatran etexilate with or without cisplatin decreased the number of TF^+^ microparticles of platelet and ID8 tumor cell origin (Figure 2D).

### Co-treatment with cisplatin and dabigatran etexilate reduces specific subpopulations of suppressor cells and increase T cell interferon-γ (IFN-γ) production in the ascites

**Figure 3:**
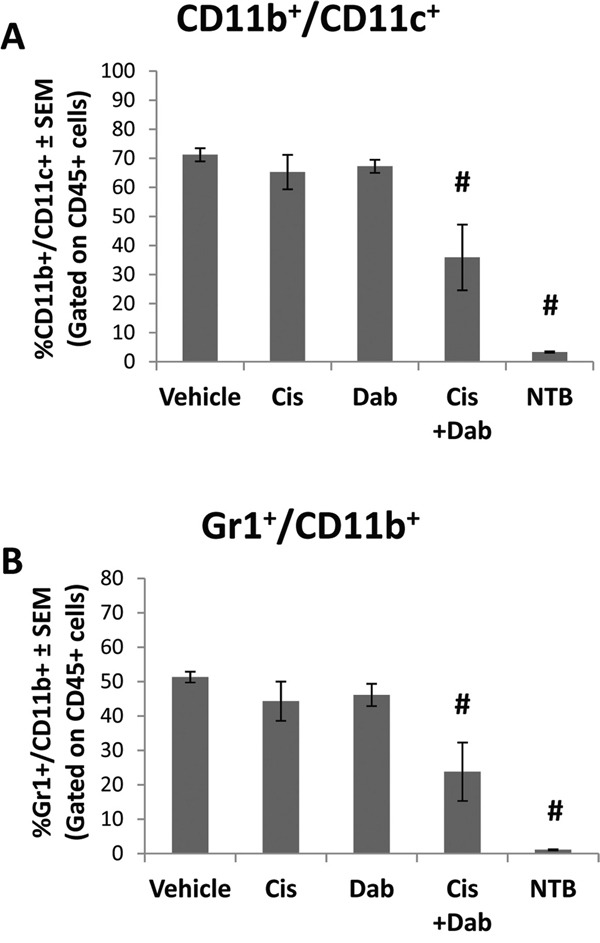
Cisplatin and dabigatran etexilate co-treatment reduces immunosuppressive cell populations Upon sacrifice, ascites was removed from ID8 tumor bearing mice and spun at 300 x g for 10 minutes to isolate the cellular component. A peritoneal lavage was performed to obtain resident cells from non-tumor bearing mice. CD45^+^ ascites cells were analyzed for the percentage of **A.** CD11b^+^/CD11c^+^ and **B.** Gr1^+^/CD11b^+^ cells by flow cytometry. n = 5-10 mice per group. * = p<0.05 and # = p<0.01 compared to control vehicle-treated tumor bearing mice or indicated groups.

The cellular component of ascites in ID8 tumor bearing mice was analyzed for inflammatory infiltrates by flow cytometry. Following treatment with dabigatran etexilate and cisplatin there were no significant differences in total CD45^+^ immune cells in the ascites. Treatment with either cisplatin or dabigatran etexilate individually resulted in only minor differences in CD45^+^ leukocyte subpopulations in the ascites of ID8 tumor bearing mice (Supplementary Table S1 and S2). However, co-treatment with both cisplatin and dabigatran etexilate significantly decreased the ascites subpopulation of CD11b^+^ CD11c^+^ cells which have been reported to be immunosuppressive myeloid-like dendritic cells [23] (Figure 3A). Co-treatment with cisplatin and dabigatran etexilate also significantly decreased the population of Gr1^+^ CD11b^+^ myeloid derived suppresser cells (MDSC), another major immunosuppressive cell type found in many tumor types (Figure 3B).

**Figure 4:**
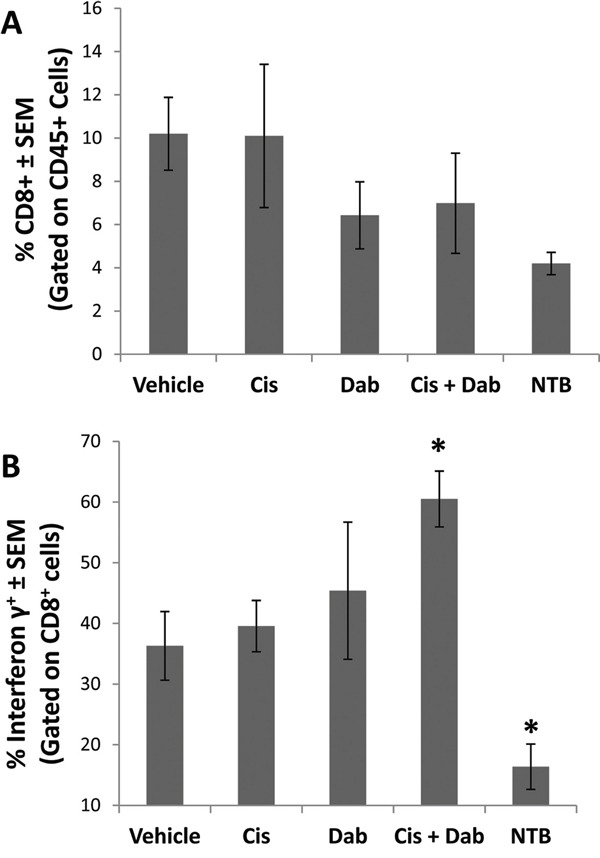
Cisplatin and dabigatran etexilate co-treatment increases CD8^+^ T cell IFN-γ production Upon sacrifice, ascites was collected from ID8 tumor bearing mice and spun at 300 x g for 10 minutes to isolate the cellular component. A peritoneal lavage was performed to obtain resident cells from non-tumor bearing mice. Ascites cells (2 x 10^6^) were treated for 4 hours at 37°C with ionomycin (500 ng/ml) and PMA (50 ng/ml) to stimulate activated T cells to produce IFN-γ in the presence of Brefeldin A to block cytokine secretion. **A.** Cells were surface stained with anti-CD8α and anti-CD45 antibodies in the presence of Brefeldin A. **B.** Cells were fixed, permeabilized and intracellularly stained with an anti-IFN-γ antibody and analyzed by flow cytometry. n = 5-10 mice per group. * = p<0.05 and # = p<0.01 compared to control vehicle-treated tumor bearing mice or indicated groups.

To further study the cellular component of the ascites, we analyzed the ability of CD8^+^ T effector cells in the ascites to produce IFN-γ, an immunostimulatory response of cytotoxic T-cells initiated once antigen-specific immunity has developed. Ascites cells were stimulated with ionomycin and PMA, and IFN-γ production by activated T cells was measured by flow cytometry. The number of CD8^+^ T-cells in the ascites of ID8 tumor bearing mice was similar in all treatment groups (Figure 4A). When stimulated, 36.3 ± 5.7% of the CD8^+^ T cells in the ascites of vehicle treated mice produced IFN-γ (Figure 4B). Treatment with cisplatin or dabigatran etexilate individually did not alter IFN-γ production. However, co-treatment with cisplatin and dabigatran etexilate significantly increased the percentage of CD8^+^ T cells producing IFN-γ to 60.5 ± 4.6%.

### Co-treatment with cisplatin and dabigatran etexilate decreases pro-tumorigenic cytokines in the ascites

**Figure 5:**
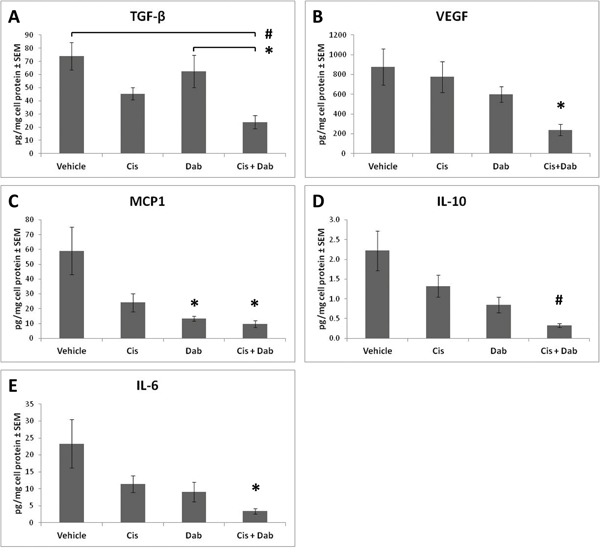
Cisplatin and dabigatran etexilate co-treatment reduces levels of pro-tumorigenic cytokines in the ascites Upon sacrifice, ascites was removed and spun at 300 x g for 10 minutes to isolate the cell free component of the ascites which was assayed for levels of **A.** TGF-β, **B.** VEGF, **C.** MCP-1, **D.** IL-10, and **E.** IL-6 by ELISA or Cytokine Bead Array. n = 5-10 mice per group. * = p<0.05 and # = p<0.01 compared to control vehicle-treated tumor bearing mice or indicated groups.

Ascites contains a dynamic reservoir of soluble components, including cytokines, chemokines and growth factors, which affect tumor cell growth. Ascites from ID8 tumor bearing mice was centrifuged to remove the cellular component and analyzed by ELISA or cytokine bead analyses using flow cytometry. Transforming growth factor beta (TGF-β) and vascular endothelial growth factor (VEGF) are both found in abundance in the ascites of ovarian cancer patients and play an important role in modulating the tumorigenicity of ovarian cancer cells [18]. Treatment with cisplatin or dabigatran etexilate individually did not alter the levels of TGF-β and VEGF in the ascites of ID8 tumor bearing mice (Figure 5); however, co-treatment with both significantly reduced levels of both TGF-β and VEGF. Dabigatran etexilate, with or without cisplatin, also reduced the levels of the proinflammatory cytokines MCP-1, IL-6 and IL-10 in the ascites of ID8 tumor bearing mice (Figure 5). In mice with similar levels of tumor burden, co-treatment with both cisplatin and dabigatran significantly reduced the levels of TGF-β, VEGF and IL-6, while both cisplatin and dabigatran individually lowered the levels of IL-10 and MCP-1 (Supplementary Figure S2) Overall, the enhanced anti-tumor efficacy with dabigatran etexilate and cisplatin co-treatment was accompanied by a decrease in immunosuppressive myeloid cell populations and pro-inflammatory cytokines in the tumor ascites.

### ID8 ovarian carcinoma cells produce pro-tumorigenic cytokines and promote the pro-tumorigenic M2 polarization of macrophages

**Figure 6:**
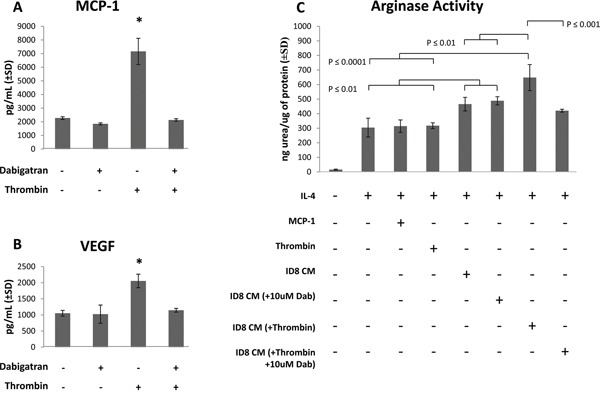
ID8 conditioned media (CM) promotes M2 polarization of IL-4 treated macrophages ID8 CM was collected after culture of ID8 cells for 48 hours in starving medium ± 0.1 Units/mL thrombin and ± 10 uM dabigatran. After centrifugation, ID8 CM was assayed for **A.** MCP-1 and **B.** VEGF. Murine RAW264.7 macrophage cells were treated with IL-4 in the presence or absence MCP-1 (3.2 ng/mL), thrombin (0.1 Units/mL) or ID8 conditioned media (10% by volume), and after 18 hours cell lysates were assayed for **C.** arginase activity (urea production). All cell cultures were assayed in at least quadruplicate. * = p<0.001 compared to indicated groups.

Conditioned media (CM) was generated from ID8 ovarian carcinoma cells in tissue culture ± 0.1 Units/mL of recombinant thrombin, with or without 10 uM dabigatran. CM was centrifuged to remove cellular debris and analyzed by ELISA or cytokine bead analyses using flow cytometry to identify any cytokines and chemokines secreted by ID8 cells. MCP-1 and VEGF were identified in CM in the absence of thrombin (Figure 6A and 6B), and levels were not affected by the addition of 10 uM dabigatran. Levels of both MCP-1 and VEGF were significantly increased by the addition of 0.1 Units/mL of thrombin and blocked by dabigatran (Figure 6A and 6B).

Because Th2 cytokines such as IL-4 have been shown to polarize macrophages to an arginase-expressing pro-tumorigenic M2 phenotype, we examined the effect of ID8 CM on the M1/M2 polarization of the murine macrophage cell line RAW264.7. As expected, IL-4 induced an M2 phenotype characterized by an increase in arginase activity (Figure 6C). The addition of ID8 CM (10% by volume), which contained high levels of MCP-1 and VEGF, resulted in a 50% increase in arginase activity over macrophages cultured with IL-4 alone. ID8 CM collected from tumor cells cultured in the presence of 0.1 Units/mL of thrombin further induced arginase activity in RAW264.7 cells. However, CM collected after the addition of dabigatran to thrombin-treated ID8 cells resulted in significantly less arginase induction in the RAW264.7 cells compared to that with CM from thrombin-treated ID8 tumor cells (Figure 6C). The addition of thrombin or recombinant MCP-1 directly to the RAW264.7 cells resulted in no further induction of arginase activity over cells treated with IL-4 alone. ID8 CM, thrombin or MCP-1 had no effect on M1 polarization (data not shown). These data suggest that thrombin induces the secretion of pro-tumorigenic cytokines, including MCP-1 and VEGF, from ID8 ovarian tumor cells, which can be blocked by dabigatran. Moreover, the thrombin-induction of cytokines from ID8 tumor cells contributes to the M2 polarization of macrophages which cannot be simply mimicked by the direct addition of thrombin or MCP-1 to macrophages.

## DISCUSSION

**Figure 7:**
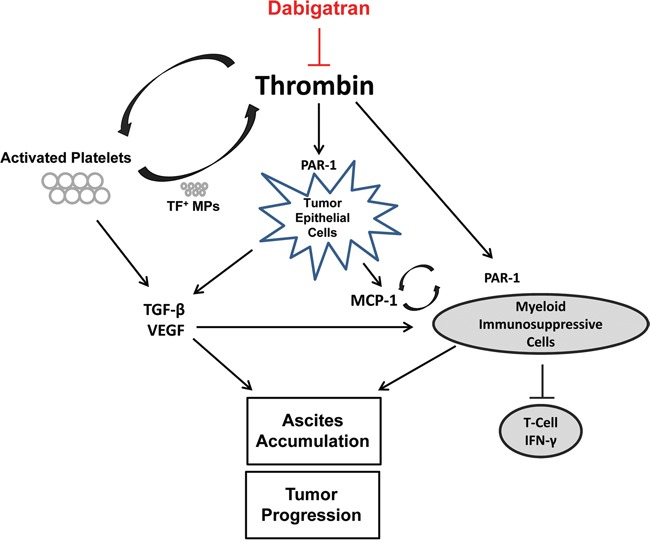
Inhibition of ovarian tumor progression by dabigatran etexilate Thrombin has many activities that promote cancer progression. Thrombin can induce TGF-β via platelet activation and PAR-1 signaling in the tumor epithelial cells. Elevated ascites levels of TGF-β, VEGF, and MCP-1 impact the recruitment and expansion of myeloid derived immunosuppressive cells, such as MDSCs and DCs. These myeloid derived cell populations, which can also be directly modulated by thrombin via PAR-1 signaling, impair T-cell and natural killer cell function contributing to the immunosuppressive microenvironment. Dabigatran etexilate directly inhibits thrombin, disrupting many of these protumorigenic pathways.

Thrombotic complications are common in patients with advanced stages of cancer and are a primary cause of death [24]. The pro-thrombotic microenvironment also directly promotes tumor growth and metastasis [25]. Common chemotherapeutic treatments can significantly increase the occurrence of thrombotic complication in patients. Therefore, we hypothesized that inhibiting thrombin may enhance the anti-tumor efficacy of a chemotherapeutic agent when treating ovarian cancer. Our results show that co-treatment with cisplatin and dabigatran etexilate, an orally administrated direct thrombin inhibitor, significantly inhibit ovarian cancer progression in the murine ID8 tumor model. Of particular importance was the novel discovery that the anti-tumor effect of co-treatment with dabigatran and cisplatin was associated with dramatic immunomodulatory effects including decreased levels of proinflammatory cytokines, reduced immunosuppressive myeloid cell populations in the ascites, and a concomitant increase in CD8^+^ T cell antitumor activity (Figure 7).

There is a well-established link between inflammation and cancer [26]. Moreover, extensive cross-talk exists between inflammation and coagulation systems, whereby inflammation not only leads to activation of coagulation, but coagulation also affects inflammatory activity [27]. Inflammation initiates the recruitment and activation of numerous immune cells which produce and release a variety of inflammatory factors, generating an autonomous loop of chronic inflammatory responses. As chronic inflammation progresses, an immunosuppressive microenvironment is induced, characterized by the accumulation of immune suppressor cells, such as MDSCs, pro-inflammatory cytokines, growth and angiogenic factors that suppress T-cell and natural killer cells activity [28]. Chronic inflammation has been shown to induce thrombin expression [29, 30] mediated by tissue factor induction by the proinflammatory cytokine IL-6 [31]. A major source of tissue factor is TF^+^ microparticles secreted by activated platelets which are potently activated by thrombin. In turn, microparticles released by activated platelets have exposed phosphatidylserine on their surface, which in conjunction with TF, are potent initiators of the conversion of prothrombin to thrombin. Similar to what has been observed in cancer patients [32] and other tumor models [22], we observed a significant increase in circulating TF^+^ microparticles in ID8 tumor-bearing mice compared to non-tumor bearing mice. Inhibition of thrombin with dabigatran etexilate completely prevented the tumor-associated increase of circulating TF^+^ microparticles in ID8 tumor-bearing mice.

Thrombin has the potential to directly modulate the immune response to the developing tumor via PAR-1 signaling. In addition to PAR-1 expression on the tumor epithelial cells themselves, PAR-1 is also abundantly expressed in the tumor microenvironment, including on infiltrating macrophages/monocytes, lymphocytes, endothelial cells, fibroblasts, and smooth muscle cells [33]. Indeed, PAR-1 expression by cells in the microenvironment drives tumor progression and metastasis. Illustrating this, ablation of PAR-1 from the tumor microenvironment, but not the tumor, has been shown to dramatically reduce tumor growth and metastasis in multiple tumor models [34, 35], in part by reducing infiltration of M2-like macrophages into the tumor [34]. PAR-1 stimulation of fibroblasts or macrophages with thrombin causes secretion of MCP-1 while at the same time enhancing monocyte/macrophage migration towards MCP-1 in a PAR-1-dependent manner [34, 36]. We demonstrated that ID8 cells produce MCP1 and that conditioned media from such cells promotes the conversion of RAW 264.7 cells to an arginase expressing M2 phenotype, while *in vivo* inhibition of thrombin with dabigatran etexilate significantly reduced levels of MCP-1 in the ascites of ID8-tumor bearing mice as well as populations of Gr-1^+^CD11b^+^ and CD11c^+^CD11b^+^ myeloid cells. Since monocyte conditioned medium has been shown to reduce chemotherapy-induced tumor cell death [34], it is possible that the reduced numbers of recruited monocytes in the tumor ascites of dabigatran-treated mice contributed to the increased cisplatin sensitivity and anti-tumor effect of dabigatran and cisplatin co-treatment.

Co-treatment with both cisplatin and dabigatran etexilate not only inhibited ID8 tumor growth in mice but also inhibited the development of malignant ascites. Malignant ascites is a reservoir of proinflammatory cytokines, chemokines, growth factors and cells which interact to affect tumor cell growth and progression by multiple mechanisms [16, 18]. A profile of cytokines in the ascites of epithelial ovarian cancer patients identified enhanced expression of many factors including angiopoietin, IL-6, IL-8, IL-10 MCP-1, and RANTES [37]. IL-10 has been shown to inhibit T helper cell production, impair dendritic cell maturation and inhibit T cell co-stimulatory molecules, suggesting that IL-10 in the ascites helps shield tumor cells from immunosurveillance [38]. In ovarian cancer, high levels of IL-6 promote tumor growth, migration, invasion [39] and facilitate chemoresistance and angiogenesis [40, 41]. High levels of both IL-6 and IL-10 expression in ascites have been associated with shorter progression-free survival, poor survival and poor initial response to chemotherapy [42]. Co-treatment with dabigatran etexilate and cisplatin reduced levels of pro-tumorigenic cytokines, specifically IL-6 and IL-10, in the ascites compared to vehicle-treated mice, dramatically augmenting the anti-tumor efficacy of cisplatin treatment. Interestingly, dabigatran etexilate treatment alone significantly reduced MCP-1 cytokine levels in the ascites of tumor-bearing mice. Incubation *in vitro* with thrombin dramatically increased the secretion of MCP-1 by ID8 cells, suggesting a possible mechanism for the reduction of MCP-1 in the ascites of mice treated with dabigatran etexilate. The dabigatran-reduction in MCP-1 levels correlated with the decreased recruitment of myeloid immunosuppressor populations in the tumor ascites as well.

Co-treatment of ID8 tumor bearing mice with cisplatin and dabigatran etexilate also significantly reduced the levels of TGF-β and VEGF in the ascites. VEGF is present at high levels in the ascites of ovarian cancer patients and plays an important role in tumor progression and dissemination by altering the permeability of the peritoneal membrane. High VEGF production in ovarian tumors is associated with increased metastatic spread and poor prognosis compared to low VEGF-secreting tumors [43]. Conversely, VEGF inhibition suppresses the formation of ascites in mice with ovarian tumors [44]. Multiple factors have been shown to increase VEGF production by ovarian cancer cells including; hypoxia, lysophosphatidic acid, matrix metalloproteinases, platelet derived growth factor, and TGF-β [16]. Thrombin was also shown to increase the secretion of VEGF directly from ID8 ovarian carcinoma cells. Blockade of TGF-β inhibits tumor spread and ascites formation via inhibition of VEGF expression in orthotopic human ovarian cancer models [45]. TGF-β release and activation is regulated by thrombin via multiple mechanisms. Thrombin-activation of platelets triggers the release of TGF-β from α-granules of platelets, and thrombin activity can also release latent TGF-β from extracellular matrix stores [22]. In a murine model of metastatic breast cancer, we have shown that inhibition of thrombin with dabigatran etexilate reduced both TGF-β levels and platelet activation [22].

Immunosuppressive cytokines in malignant ascites have been shown to impair the polyfunctional response of T cells, particularly the generation of IFN-γ by cytotoxic CD8^+^ T cells [46]. The presence of tumor-infiltrating CD8^+^ T cells in primary tumors has been associated with prolonged disease-free survival and overall survival of ovarian cancer patients [47, 48]. Cytokines such as TGF-β and VEGF have been shown to promote the expansion of Gr1^+^CD11b^+^ MDSCs which suppress tumor infiltrating T cells and natural killer cells [49, 50]. Immunosuppressive CD11c^+^CD11b^+^ dendritic cells found in malignant ascites secrete IL-10 and TGF-β resulting in inhibition of CD8^+^ T cell function and enhanced generation of CD4^+^CD25^+^FoxP3^+^ regulatory T cells [23]. Importantly, we show that the anti-tumor effect of co-treatment with cisplatin and dabigatran etexilate correlates with reduced accumulation of immunosuppressive monocyte subpopulations and an associated increase in IFN-γ production from CD8^+^ T cells.

On-going clinical trials, such as the CANVAS trial [51], are studying the efficacy of dabigatran etexilate and other oral anticoagulants in preventing VTE in cancer patients, but none are directly investigating if anti-coagulation therapy has a direct effect on cancer progression and outcomes. Our results indicate that dabigatran etexilate in combination with standard frontline chemotherapeutics, such as platinum-base therapy, may be a more effective treatment than chemotherapeutics alone, but safety and efficacy studies need to be performed to confirm this hypothesis. However, unlike warfarin, Pradaxa requires no monitoring and has an FDA approved direct antidote, (idarucizumab) Praxbind®, available [52]. Additionally, thrombin inhibition with dabigatran etexilate would likely help alleviate the thrombotic complications often associated with malignant cancers.

Another interesting possibility would be the investigation of molecules or complexes that contribute to thrombosis but do not effect hemostasis, potentially achieving some of the benefits of thrombin inhibition without the potential for bleeding complications [53]. In a recent review, Geddings and Mackman highlight several such factors [53]. The protease FXII is part of the intrinsic coagulation cascade and has been shown to directly increase fibrin fiber density independent of thrombin activation [54]. Interestingly, FXII-deficient mice showed reduced thrombosis in arterial damage models without any increase in tail vein bleeding times [55]. Moreover, neutrophil extracellular traps (NETS) are release by activated neutrophils and have been shown to enhance arterial thrombosis by providing a negatively-charged surface for the activation of FXII [56], inactivating tissue factor pathway inhibitor [57] and by binding platelets and red blood cells [58]. Inhibition of these factors as they pertain to tumor growth and metastasis warrants further investigation.

These results suggest that co-treatment with the thrombin inhibitor dabigatran etexilate and low dose cisplatin significantly inhibit ovarian tumor growth and ascites development by modulating the tumor microenvironment in several notable and novel ways: 1) co-treatment significantly reduced Gr1^+^CD11b^+^ and CD11c^+^CD11b^+^ myeloid derived suppresser cell populations; 2) decreased levels of multiple pro-inflammatory cytokines including IL-10, IL-6, TGF-β and VEGF; and 3) increased IFN-γ production by CD8^+^ T-cells. This reversal of the proinflammatory and immunosuppressive microenvironment inherent to malignant tumors by co-treatment with dabigatran etexilate and cisplatin highlights a hereto unidentified aspect of thrombin's involvement in cancer progression and presents a potential target for treatment of malignant tumors.

## MATERIALS AND METHODS

### Animals

Female C57/Bl6 mice were obtained from Charles Rivers/NCI. Protocols for the use of animals in these studies were reviewed and approved by the Institutional Animal Care and Use Committee of the Lankenau Institute for Medical Research in accordance with the current US Department of Agriculture, Department of Health and Human Service regulations and standards.

### Cell culture

The luciferase expressing ID8-luc mouse ovarian carcinoma cell line was kindly provided by Dr. Janet Sawicki of the Lankenau Institute of Medical Research. Cells were cultured in DMEM supplemented with 4% fetal bovine serum, 1x insulin/transferrin/sodium selenite media supplement (Corning) and 1x Penicillin/Streptomycin (Cellgro).

### *In vivo* ID8 tumor model

Female C57/Bl6 mice were intraperitoneally (i.p.) injected with 1.0 x 10^6^ ID8-luc cells. To monitor tumor spread throughout the peritoneal cavity, mice were imaged for bioluminescence. When tumor bioluminescence values were approximately 5.0 x 10^5^ photons/sec/cm^2^ treatment was initiated. Cisplatin (1 mg/kg) was i.p. injected once weekly with or without dabigatran etexilate administration. Mice were dosed with dabigatran etexilate by oral gavage twice daily (80 mg/kg) Monday through Friday and placed on dabigatran chow (10 mg/g chow) over the weekends. Collected ascites was spun at 300x g for 5 minutes to precipitate cells. The supernatant was removed and frozen at -80°C while the remaining cells in the pellet were analyzed by flow cytometry or frozen at -80°C.

### Tail clip assay

Mice were either dosed with dabigatran etexilate by oral gavage (80 mg/kg) one time or placed on dabigatran chow (10 mg/g chow) for 48h and then anesthetized via IP administration of ketamine. Thirty minutes after oral gavage the distal portion of the tail was transected at a diameter of 2.5 mm and placed in a conical tube containing 14 ml of saline at 37°C. The tail was allowed to bleed into the tube of saline for 5 min. The injured tail was move to a fresh tube of saline, and blood was collected for 10 min. Quantitative assessment of blood loss was determined by measuring total hemoglobin by absorbance at 575 nm as previously described [59]. Quantitative assessment of hemoglobin content was converted to total blood loss (μl) by using appropriately generated standard curves.

### Analysis of circulating platelets

Blood was collected from the vena cava using a syringe pre-loaded with citrate-dextrose. To count platelets, 50 μl of whole blood was diluted 1:20 with 1% ammonium oxalate monohydrate and set to shake at room temperature for 10 min to lyse red blood cells. Platelets were manually counted using a hemocytometer. The remaining blood was sequentially centrifuged to separate platelet poor plasma (PPP) and frozen at -80°C for later analysis of microparticles. Platelets were assessed for activation by flow cytometry. Equal numbers of viable platelets were stained with PE-Cy7 conjugated anti-CD41 (eBioscience) and PE-conjugated anti-CD62P (P-selectin, eBioscience). Flow-cytometric data were acquired on a BD FACSCanto II and analyzed using FACSDiva software (BD Biosciences).

### Flow cytometry analysis of tissue factor positive microparticles

For flow-cytometric analysis, 15 μl of PPP was diluted with 85 μl of Annexin V binding buffer (eBioscience) and stained with APC-conjugated Annexin V (eBioscience) and goat polyclonal anti-mouse TF antibody (RD systems) followed by incubation with a FITC-conjugated anti-goat IgG secondary antibody (Novus Biologicals) [60]. After incubation, samples were diluted with 1.5 ml of Annexin V binding buffer. Analysis of samples was conducted using a BD FACSCanto II and analyzed using FACSDiva software. Microparticles were identified by size (forward scatter) and annexin V binding. Annexin V positive events between 0.5 and 1 μm were counted as microparticles. Microparticles smaller than 0.5 μm we not included in the analysis due to difficulties distinguishing small microparticles from background noise. The size of the gate was adjusted using fluorescent microbeads measuring 0.5, 1.0 and 3.0 μm (Molecular Probes). Thresholds for TF-positive microparticles were set from annexin V binding positive microparticles. To identify the cell origin of microparticles, annexin V^+^ and TF^+^ microparticles in PPP were also stained with PE-Cy7-conjugated anti-CD41 (eBioscience) as a platelet marker, APC-Cy7-conjugated anti-CD45 (BD Pharmingen) as a leukocyte marker, PerCP-Cy5.5 conjugated anti-CD31 (BD Pharmingen) as an endothelial marker, and PE-conjugated anti-CD326 (BD Pharmingen) as a marker for ID8 tumor epithelial cells.

### Flow cytometry analysis of ascites

Ascites cell pellets were incubated with 5 ml of red cell lysis buffer (0.17 M Tris-HCL, 0.16 M NH_4_Cl) for 5 min. Cells were spun down and resuspended in FACS buffer (1.5% heat inactivated FBS, 0.2% NaN_3_ in PBS). Equal numbers of viable cells were stained with PE-conjugated anti-CD11b, PE-Cy7-conjugated CD-11c, FITC-conjugated anti-Gr-1, and APC Cy7-conjugated anti-CD45 (all from eBiosciences). Flow-cytometric data were acquired on a BD FACSCanto II and analyzed using FACSDiva software (BD Biosciences). Viable cells were gated based on forward and side scatter profiles.

### Intracellular IFN-γ staining

Ascites cell pellets were incubated with 5 ml of red cell lysis buffer for 5 min and then equal numbers of cells (2.0 x 10^6^) were resuspended in Iscove's Modified Dulbecco's culture media (Gibco) supplemented with 10% heat inactivated fetal calf serum, 1% Glutamax (Gibco), 0.5% gentamycin and 50 μM 2-mercaptoethanol. Cells were treated for 4 hours at 37°C with ionomycin (500 ng/ml) and phorbol myristate acetate (PMA) (50 ng/ml) to stimulate activated T cells to produce IFN-γ in the presence of Brefeldin A (eBioscience) to block cytokine secretion. Cells were surface stained with PE-Cy7-conjugated anti-CD8α and APC-Cy7-conjugated anti-CD45 in the presence of Brefeldin A. Cells were fixed, permeabilized, and intracellularly stained with APC-conjugated anti-IFN-γ (eBioscience). Flow-cytometric data were acquired on a BD FACSCanto II and analyzed using FACSDiva software (BD Biosciences). Viable cells were gated based on forward and side scatter profiles.

### Cytokine analyses

Ascites samples were spun at 300 x g for 10 minutes to pellet cells. Ascites supernatants were collected and analyzed for TGF-β, monocyte chemoattractant protein-1 (MCP-1), IL-6, and IL-10 using TGF-β and Mouse Inflammation Cytometric Bead Array reagents (BD Biosciences, San Jose, CA) and flow cytometry as per the manufacturer's protocol. VEGF cytokine levels were analyzed using the mouse VEGF Quantikine ELISA (R&D systems) as per the manufacturer's instructions.

### RAW 264.7 M1/M2 polarization assay

RAW 264.7 cells were plated at 5x10^5^ cells per well (96 well plate) in DMEM + 10% heat inactivated FBS for 8 hours. Cells were then washed once with RPMI + 1% heat inactived FBS and then treated overnight in 100 ul of same media containing either LPS (100 ng/mL) to promote M1 polarization or IL-4 (5 ng/mL) to promote M2 polarization. Additional treatments were added as indicated. To quantify M1 polarization, a Greiss assay was performed on 50 ul of the treatment media to measure nitrite levels. To measure M2 polarization, arginase activity was measure in cell lysates as previously described [61]. Briefly, cells were lysed in 100 ul of 0.1% Triton X-100, 25 mM Tris HCl and Halt protease/phosphatase cocktail, pH 8.0 (Thermo Scientific). Half of the lysate was diluted 1:2 with lysis buffer while the other half was used to determine protein concentration. To 100 ul of the diluted lysate, 10 ul of 10 mM MnCl_2_ was added and the samples were incubated at 55°C for 10 minutes to activate the enzyme. Arginine hydrolysis was measured by incubating the heated lysates with 100 ul of 0.5 M L-arginine (pH 9.7) at 37°C for 2 hours. The reaction was stopped with 800 ul of H_2_SO_4_ (96%)/H_3_PO_4_ (85%)/ H_2_O (1:3:7, v/v/v). Urea concentration was measured at 540 nm after the addition of 40 ul of a-isonitrosopropiophenone (dissolved in 100% ethanol) followed by heating to 105°C for 90 minutes. The rate of urea production was used as an index for arginase activity.

### Generation of ID8 conditioned media

ID8 cells were plated at 5 x10^4^ cells per well in a 24 well plate in DMEM supplemented with 4% fetal bovine serum, 1x insulin/transferrin/sodium selenite media supplement (Corning) and 1x Penicillin/Streptomycin (Cellgro) and allowed to plate down overnight. The next morning the cells were incubated for 4 hours in starving media (DMEM + 1X penicillin/streptomycin but no serum or other supplements). The starving media was replaced with treatment media (starving media ± 0.1 Unit/mL thrombin (Sigma) and ± 10 μM dabigatran) and was incubated for 48 hours. After 48 hours media was collected and spun at high speed to remove cellular debris.

### Statistics

All *in vivo* experiments were carried out using multiple animals (n = 4-10 per experimental group). All *in vitro* experiments were performed in at least triplicate, and data compiled from 2-3 separate experiments. All analyses were done using a 1-way ANOVA with a Tukey test for statistical significance or Students T-tests.

## SUPPLEMENTARY MATERIALS FIGURES AND TABLES




