# Supplementary material for: Thrombin inhibition and cisplatin block tumor progression in ovarian cancer by alleviating the immunosuppressive microenvironment
Source: Oncotarget. 2016 Nov 11;7(51):85291–305. doi: 10.18632/oncotarget.13300 (PMC5356737; doi:10.18632/oncotarget.13300)
Supplement: Supplementary file 1 [file oncotarget-07-85291-s001.pdf]

Thrombin inhibition and cisplatin block tumor progression in ovarian cancer by alleviating the immunosuppressive microenvironment

SUPPLEMENTARY FIGURES AND TABLES

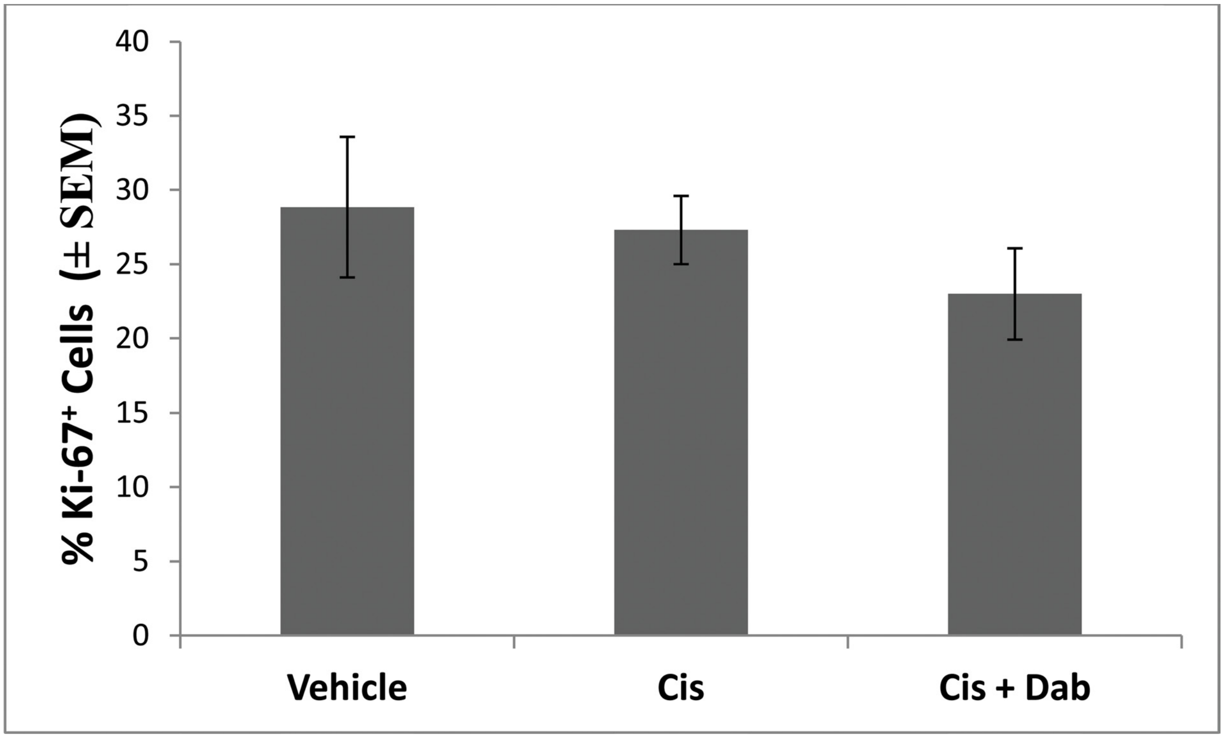

Supplementary Figure S1: Treatment with cisplatin and dabigatran etexilate does not affect ID8 tumor cell proliferation. ID8 tumors on the peritoneal wall were fixed and sectioned for histology. Sections were stained with Ki-67, a marker of cell proliferation. N = 5-7 mice per group with 1000 tumor epithelial cells counted per sample. Values expressed as Ki-67$^+$ cells/total tumor epithelial cells x 100.

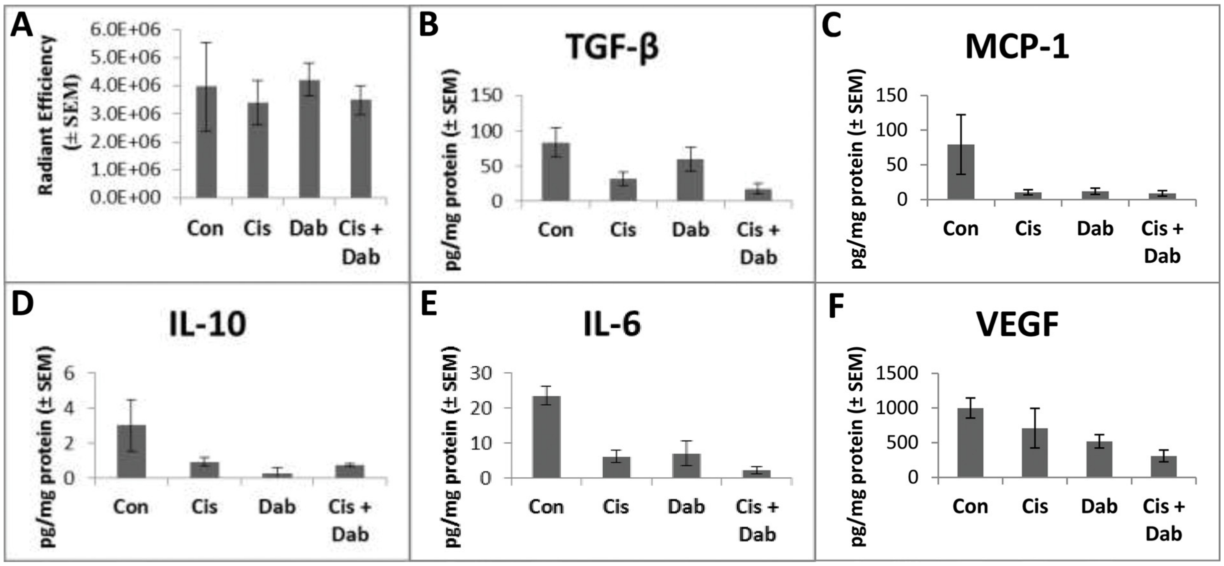

Supplementary Figure S2: Cisplatin and dabigatran etexilate co-treatment reduces levels of pro-tumorigenic cytokines in the ascites in mice with similar tumor burden. Mice with similar ID8 tumor burden were compared **A.** Upon sacrifice, ascites was removed and spun at 300 x g for 10 minutes to isolate the cell free component of the ascites which was assayed for levels of **B.** TGF-β, **C.** MCP-1, **D.** IL-10, **E.** IL-6, and **F.** VEGF by ELISA or Cytokine Bead Array. n = 5-10 mice per group.

Supplementary Table S1: Cellular analysis of malignant ascites (cell number x 10^4)

See Supplementary File 1

Supplementary Table S2: Cellular analysis of malignant ascites. (Percentages)[a]

See Supplementary File 1
